# Prevalence of urinary tract infections in pregnant women and antimicrobial resistance patterns in women in Riyadh, Saudi Arabia: a retrospective study

**DOI:** 10.1186/s12879-024-09385-y

**Published:** 2024-05-18

**Authors:** Yasmin Barnawi, Ahlam Alghamdi, Alnada Ibrahim, Lina Al-Anazi, Ghada Alhumaida, Reema Alotaibi, Mohammad Khan, Dareen Baz, Mohammed Alraey, Afrah Alkazemi, Hajar Alqhatani, Hadeel Waggas

**Affiliations:** 1 Department of Pharmacy Practice, College of Pharmacy, Princess Nourah Bint Abdulrahman University, P.O. Box 84428, Riyadh, 11671 Saudi Arabia; 2 Pharmacy Department, Prince Sultan Military Medical City, P.O. Box 7897, Riyadh, 11159 Saudi Arabia; 3 College of Pharmacy, Princess Nourah Bint Abdulrahman University, P.O. Box 84428, Riyadh, 11671 Saudi Arabia; 4 Department of Microbiology, King Abdullah Bin Abdul-Aziz University Hospital, Princess Nourah Bint Abdulrahman University, P.O. Box 84428, Riyadh, 11671 Saudi Arabia; 5 College of Medicine, AlFaisal University, P.O. Box 50927, Riyadh, 11533 Saudi Arabia; 6 Infectious Diseases Department, King Abdullah bin Abdulaziz University Hospital, P.O. Box 84428, Riyadh, 11671 Saudi Arabia; 7 Department of Pharmacy Practice, College of Pharmacy, Kuwait University, P.O. Box 5969, Safat, 13060 Kuwait; 8 Department of Pharmaceutical Care, Ministry of National Guard Health Affairs, P.O. Box 22490, Riyadh, 11426 Saudi Arabia; 9 King Abdullah International Medical Research Center, P.O. Box 3660, Riyadh, 11481 Saudi Arabia; 10 King Saud Bin Abdul-Aziz University for Health Sciences, P.O. Box 3660, Riyadh, 11481 Saudi Arabia; 11 Department of Obstetrics and Gynecology, King Abdullah bin Abdulaziz University Hospital, Princess Nourah Bint Abdulrahman University, P.O. Box 84428, Riyadh, 11671 Saudi Arabia

**Keywords:** Prevalence, Pregnant, Susceptibility, Urinary tract pathogens, Saudi Arabia, Prenatal education

## Abstract

**Background:**

Urinary tract infections (UTIs) are one of the most common health problems worldwide and mainly affect women. This study aimed to evaluate the prevalence of UTIs in pregnant women and determine the antimicrobial resistance patterns of bacterial pathogens isolated from pregnant and nonpregnant women in Riyadh, Saudi Arabia.

**Methods:**

This retrospective cohort study was conducted at an academic medical center in Riyadh, Saudi Arabia, from January to June 2022. The study included all urine cultures performed for adult women during the study period. We excluded urine culture performed for women on antibiotics prescribed for any infection, children, and men. Using the SPSS (version 27) package, descriptive statistics and chi-square tests were used to analyze the data, and *p* < 0.05 was considered to indicate statistical significance.

**Results:**

A total of 2,418 urine cultures performed during the study period were included (985 and 1,433 for pregnant and nonpregnant women, respectively). The overall prevalence of UTIs in pregnant women was 5% (95% CI 3.6–6.4); 10 (1%) women were symptomatic, and 40 (4%) women were asymptomatic. Of the entire cohort, 244 (10.1%) women were diagnosed with UTIs based on bacterial cultures. The predominant bacteria in both pregnant and nonpregnant women were *Escherichia coli* (134, 54.9%), followed by *Klebsiella pneumoniae* (48, 19.6%).

The antibiotic susceptibility criteria for *Escherichia coli* and *Klebsiella pneumoniae* were as follows: nitrofurantoin (94% and 18.8%, respectively), amoxicillin-clavulanic acid (82.8% and 70.8%, respectively), ciprofloxacin (65.7% and 83.3%, respectively), trimethoprim-sulfamethoxazole (65.7% and 79.2%, respectively) and cephalothin (47% and 68.8%, respectively).

**Conclusion:**

Compared to the findings of other similar studies, the prevalence of UTIs was lower in pregnant women. This may be because the patient population was composed of healthy and educated women who received prenatal education and underwent prenatal assessment as per institutional guidelines. Nitrofurantoin and amoxicillin-clavulanic acid are recommended for use as an empirical therapy for UTIs in pregnant and nonpregnant women because bacteria have the least amount of resistance to these drugs.

## Background

Urinary tract infections (UTIs) are among the most common bacterial infections worldwide and commonly affect women [1]. Approximately 50 to 60% of women have at least one UTI in their lifetime [2]. One-third of women are diagnosed with UTIs before the age of 24 years, and one-half develop at least one UTI by the age of 35 years [3]. There is a greater prevalence of UTIs in pregnant women than in nonpregnant women. UTIs represent a major health problem in Saudi Arabia; they account for 10% of all infections in the country and are considered the second most common reason for emergency department admissions [4].

The global prevalence of UTIs in pregnant women is 23.9%. The reported prevalence of UTIs is as follows: Kenya (59%), Nepal (23.34%), Somaliland (16.4%), Ethiopia (23%), Cameroon (71.43%), Lucknow (45.32%), India (60.7%), Nigeria (55%), Egypt (29%), Iran (8.7%), and Sudan (70.9%) [2, 5–14].

Three published cross-sectional studies were conducted in 2013, 2016, and 2019 in different regions of Saudi Arabia, with reported incidences of 20%, 6.9%, and 53.5%, respectively, in pregnant women [15–17]. There is wide variation in the reported prevalence of UTIs in pregnant women, which can be attributed to geographic location and various factors, including increasing age, the number of births, personal hygiene, and socioeconomic status [2, 18]. *Escherichia coli* (*E. coli)*, followed by *Klebsiella pneumoniae* (*K. pneumoniae*), are the leading causes of UTIs globally [16–18]. The World Health Organization (WHO) and local guidelines recommend the use of nitrofurantoin and cotrimoxazole as first-line therapies. As antimicrobial susceptibility patterns change rapidly, new resistant strains are emerging, and updates to these guidelines are needed periodically [4].

UTIs are common in women, but the available data on the prevalence of UTIs in pregnant women are limited by small sample sizes and wide variations in the reported prevalence. The prevalence of UTIs in pregnant women living in the capital city with a dense population has not been investigated in Saudi Arabia. In addition, regular updates on antimicrobial resistance in women (pregnant and nonpregnant) are highly important. Thus, investigating the epidemiology of UTIs (prevalence and antimicrobial resistance) is fundamental for health care providers to guide optimal management and minimize UTI-associated complications. This study aimed to determine the prevalence of UTIs in pregnant women and antimicrobial resistance patterns in pregnant and nonpregnant women in the capital city of Riyadh, Saudi Arabia.

## Methods

### Study design

This was a retrospective observational cohort study conducted at King Abdullah bin Abdul-Aziz University Hospital (KAAUH) in Riyadh, Saudi Arabia, between January and June 2022. KAAUH is a 406-bed teaching hospital in Riyadh, Saudi Arabia, that aims to be accredited as a center for women’s health and pediatric and adolescent health. KAAUH provides health care services to employees and students of the hospital affiliated university and their families, as well as employees of KAAUH and their families. Institutional prenatal care includes but is not limited to urine screening at the first prenatal visit or prenatal education classes for pregnant women.

### Study participants and data collection

The urine culture data for adult women were extracted from the system. As per institutional guidelines, urine cultures were performed for all pregnant women in the first trimester as part of the screening program. Additionally, urine cultures were performed for women (pregnant and nonpregnant) who had signs and symptoms of UTIs, those who underwent a urinary procedure, and those who were critically ill.

Clinical data were collected from electronic medical records and identified for further review. Relevant information, including demographic data, baseline comorbidities, obstetrical characteristics, antimicrobial therapy regimens, urine culture results, urine analysis information and susceptibility data, was collected. UTIs were diagnosed using midstream urine culture. The data were validated by the individuals in the research group during weekly meetings.

### Urine culture, bacterial identification and antimicrobial susceptibility testing

Urine cultures were generated according to standard microbiological methods based on colony count, the number of organisms grown in culture, the use of rapid biochemical tests, and the use of automated confirmatory tests [8]. Briefly, 1 µL of urine was inoculated onto a biplate media consisting of sheep blood agar and MacConkey agar using a quantitative streak method. After 24 h of incubation, colonies of uropathogens were identified based on colony morphology, growth patterns, biochemical tests, and Gram staining. Colony counts were performed, and confirmatory identification and antibiotic susceptibility tests were completed using a Vitek2 instrument system (BioMérieux, France) with 0.5 McFarland standard inoculum as per the manufacturer’s recommendations. Antibiotic susceptibility tests were based on Clinical and Laboratory Standards Institute (CLSI) breakpoints and panels. The presence of extended spectrum beta-lactamase (ESBL) resistance in isolated uropathogens was based on the Vitek2 instrument and resistance to third-generation cephalosporins-ceftazidime and/or ceftriaxone.

### Study outcomes and definitions

The primary objective was to assess the prevalence of symptomatic and asymptomatic UTIs in pregnant women. For the secondary outcome, the antimicrobial susceptibility pattern of the bacterial pathogens was assessed based on the urine culture data collected from pregnant and nonpregnant women at KAAUH in Riyadh, Saudi Arabia.

UTIs were defined as the presence of pathogenic microorganisms in the genitourinary tract with associated signs and symptoms of infection [2].

Asymptomatic bacteriuria (ASB) was defined as the presence of one or more species of bacteria growing in the urine at specified quantitative counts (≥ 10^5^ colony-forming units [CFUs]/mL or ≥ 10^8^ CFUs/mL), irrespective of the presence of pyuria, in the absence of signs or symptoms attributable to urinary tract infection (UTI) [19].

Immunosuppressant agents, such as calcineurin inhibitors (tacrolimus and cyclosporine), antiproliferative agents (mycophenolate mofetil, mycophenolate sodium, and azathioprine), mammalian targets of rapamycin inhibitors (sirolimus and everolimus), corticosteroids (prednisone), and T-cell depleting monoclonal antibodies (alemtuzumab), were defined as therapeutic agents that inhibit or decrease the intensity of the immune response in the body [20].

### Sample size calculation

For prevalence, a minimum required sample size of 350 was estimated based on a 95% confidence interval, a 5% margin of error, and an estimated UTI incidence of 53.6% in pregnant women in a previous study conducted in Wadi Addawser, Saudi Arabia [17].

### Statistical analysis

The data were analyzed by Statistical Package for Social Sciences (SPSS) version 27.0 (IBM, USA). Descriptive statistics were determined to present the findings. Categorical variables are presented as frequencies and percentages. Continuous data are displayed as medians and interquartile ranges (IQRs). For prevalence, the proportion and 95% confidence interval were determined.

## Results

**Fig. 1:**
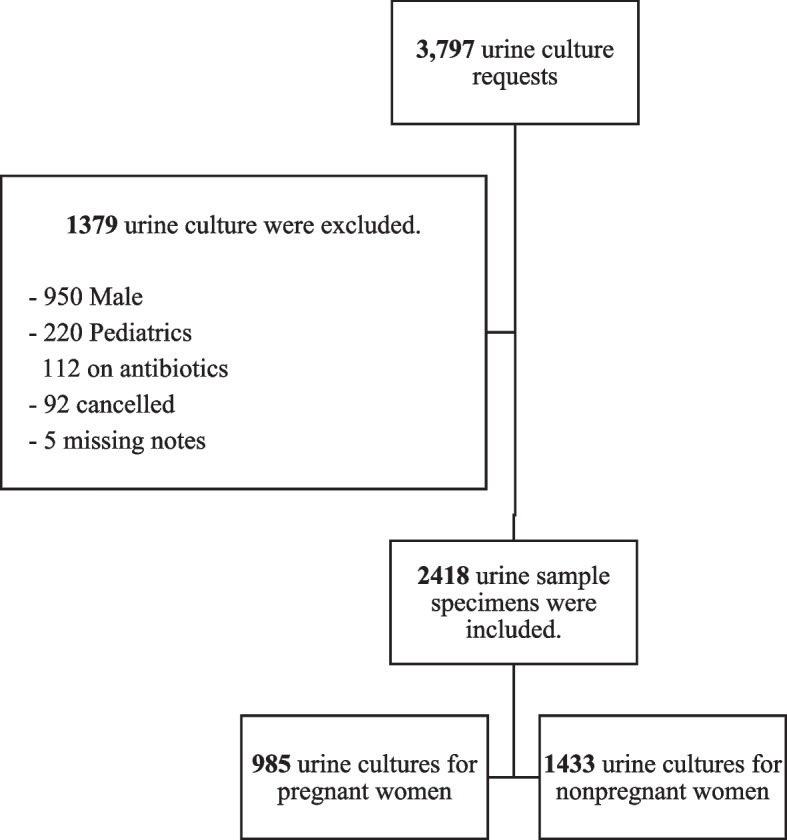
Flowchart of the urine culture performed during the study period were extracted from the system

A total of 2,418 urine cultures (985 and 1,433 for pregnant and nonpregnant women, respectively) performed during the study period were included in the analysis (Fig. 1).

In this study, the median age of the women who had urine cultures performed was 36 [29–47] years. Of the included women, 94% were outpatients. The patients tended to be healthy. The baseline characteristics are summarized in Table 1.

**Table 1:** Baseline characteristics of urine cultures of pregnant and nonpregnant women in an academic medical center in Riyadh, Saudi Arabia, from January to June 2022

**Variables**	**Pregnant (*n* = 985)**	**Nonpregnant (*n* = 1433)**	**Total (*n* = 2418)**
**Age, median [IQR]**	31 [27–36]	43 [33–59]	36 [29–47]
**BMI, median [IQR]**	29.49 [25.7–33.6]	28.47 [24.7–33.6]	28.87 [25.1–33.6]
**Heart disease**	6 (0.6)	118 (8.2)	124 (5.1)
**Diabetes**	91 (9.2)	339 (23.7)	430 (17.8)
**Hypertension**	10 (1.0)	372 (26.0)	382 (15.8)
**Chronic kidney disease**	0	39 (2.7)	39 (1.6)
**Liver disease**	1 (0.1)	10 (0.7)	11 (0.5)
**Malignancy**	0	24 (1.7)	24 (1.0)
**Respiratory disease**	41 (4.2)	211 (14.7)	252 (10.4)
**On immunosuppressant**	1 (0.1)	137 (9.6)	138 (5.7)
**History of recurrent UTI**	0	46 (3.2)	46 (1.9)
**Catheter**	1 (0.1)	14 (1.0)	15 (0.6)
**Hospitalized patients**	0	146 (10.2)	146 (6.0)
**ICU at the index of culture**	0	29 (2.0)	29 (1.2)

Variables are expressed as n (%) unless indicated otherwise

*BMI* Body mass index, *CrCl* Creatinine clearance, *WBC* White blood cell

The overall prevalence of UTIs in pregnant women was 5% (95% CI 3.6–6.4); 10 (1%) women were symptomatic, and 40 (4%) women were asymptomatic. Notably, some pregnant women might have more than one urine culture performed during pregnancy (985 urine cultures were performed for 802 pregnant women; screening and diagnosis). The obstetric and clinical characteristics are shown in Table 2.

**Table 2:** Obstetric and clinical characteristics of pregnant women attending antenatal care at an academic medical center in Riyadh, Saudi Arabia, from January to June 2022 (*n* = 802)

**Variable**	**Value, n (%)**
**Urine culture requests during gestational period**	
**First trimester**	648 (65.8)
**Second trimester**	203 (20.6)
**Third trimester**	134 (13.6)
**Gravida**	
**Primigravida**	190 (24)
**Multigravida**	612 (76)
**History of abortion**	112 (14)
**History of premature labor**	18 (2.2)
**Marital status**	
**Married**	802 (100)
**Single**	0 (0)
**History for 1 year**	
**Confirmed positive urine culture**	58 (7)
**Confirmed UTI**	48 (5.9)
**Recurrent UTI during pregnancy**	11 (1.4)

Among the 2418 urine cultures performed (for pregnant and nonpregnant women), 244 (10.1%) showed bacterial growth. Among the total isolates, the gram-negative bacteria (196 (80.3%)) were more prevalent than the gram-positive bacteria (42 (17%)). Among the isolates, the predominant bacterial *uropathogen* in both pregnant and nonpregnant women *was E. coli* (134, 54.9%), followed by *K. pneumoniae* (48, 19.6%) (Table 3).

**Table 3:** Bacterial pathogens isolated from urine of pregnant and nonpregnant women in academic medical center in Riyad, Saudi Arabia from January to June 2022 (*n* = 244)

**Bacteria isolates**	**Pregnant, 48 (19.7)**	**Nonpregnant, 196 (80.3)**	**Total, 244 (10.1)**
*E. coli*	19 (39.6)	115 (58.7)	134 (54.9)
*K. pneumoniae*	14 (29.2)	34 (17.3)	48 (19.6)
*Streptococcus agalactiae*	15 (31.3)	19 (9.7)	34 (13.9)
Others	0	28 (14.3)	28 (14.3)

Variables are expressed as n (%) unless indicated otherwise

Candida spp. accounted for 4 isolates in nonpregnant patients and zero in pregnant patients

*E. coli* was resistant to ampicillin (55.2%), cephalothin (31.3%), trimethoprim-sulfamethoxazole (34.3%), and ciprofloxacin (27.6%), and a greater percentage of susceptibility was observed for nitrofurantoin (94%), gentamicin (92.5%), and amoxicillin-clavulanic acid (82.8%).

All *K. pneumoniae* isolates were resistant to ampicillin (100%) due to an intrinsic resistance mechanism, and 33.3% were resistant to nitrofurantoin. The antibiotic susceptibility rates for the other agents tested were as follows: gentamicin (95.8%), trimethoprim-sulfamethoxazole (79.2%), amoxicillin-clavulanic acid (70.8%), ciprofloxacin (83.3%) and cephalothin (68.8%) (Table 4).

**Table 4:** Antimicrobial susceptibility of Gram-positive and Gram-negative bacteria isolated from urine of pregnant and nonpregnant women in academic medical center in Riyad, Saudi Arabia from January to June 2022 (n = 244)

**Bacteria Isolates**			** Antimicrobial susceptibility testing**						
**Gram-negative, *n* = 198 (81%)**									
		**ESBL**	**AMP**	**CEF**	**NFN**	**GN**	**STX**	**CIP**	**Amox/Clav**
***E.coli*, 134 (67.7)**		**22 (16.4)**							
	***S***		53 (39.6)	63 (47)	126 (94)	124 (92.5)	88 (65.7)	88 (65.7)	111 (82.8)
	***I***		7 (5.2)	29 (21.6)	5 (3.7)	1 (0.7)	0	9 (6.7)	12 (9.0)
	***R***		74 (55.2)	42 (31.3)	3 (2.2)	9 (6.7)	46 (34.3)	37 (27.6)	11 (8.2)
***K.pneumoniae*, 48 (24.2)**		**7 (14.6)**							
	***S***		0	33 (68.8)	9 (18.8)	46 (95.8)	38 (79.2)	40 (83.3)	34 (70.8)
	***I***		0	1 (2.1)	23 (47.9)	0	0	7 (14.6)	6 (12.5)
	***R***		48 (100)	14 (29.2)	16 (33.3)	2 (4.2)	10 (20.8)	1 (2.1)	8 (16.7)
**Others, 16 (8.1)**		0							
	***S***		14 (87.5)	13 (81.3)	13 (81.3)	16 (100)	16 (100)	11 (68.8)	15 (93.8)
	***I***		0	0	0	0	0	1 (6.3)	0
	***R***		2 (12.5)	3 (18.8)	3 (18.8)	0	0	4 (25)	1 (6.3)
**Gram-positive isolate, *n* = 42 (17.2%)**									
***Streptococcus agalactiae*, 34 (81%)**		intrinsically susceptible to penicillin							
**Others, 8 (19)**			**AMP**	**NFN**	**BP**	**EM**	**OXC**	**CIP**	**VAN**
	***S***		8 (100)	6 (75)	6 (75)	4 (50)	6 (75)	5 (62)	8 (100)
	***I***		0	2 (25)	2 (25)	1 (12.5)	0	2 (25)	0
	***R***		0	0	0	3 (37.5)	2 (25)	1 (12.5)	0

*AML* Ampicillin, *BP* Benzyl penicillin, *CEF* Cephalothin, *CIP* Ciprofloxacin, *E. coli Escherichia coli*, *EM* Erythromycin, *ESBL* Extended spectrum beta-lactamase, *GN* Gentamicin, *I* Intermediate, *K. pneumoniae Klebsiella pneumoniae*, *NFN* Nitrofurantoin, *OXC* Oxacillin, *R* Resistance, *S* Sensitive, *STX* Trimethoprim-sulfamethoxazole, *VAN* Vancomycin

*Streptococcus agalactiae* strains were routinely susceptible to penicillin, and no antibiotic susceptibility tests were performed or reported by the Microbiology Laboratory.

*E. coli* was the most common ESBL producer, with 22 isolates (16.4%), followed by *K. pneumoniae*, with 7 isolates (14.6%); none of the other gram-negative bacteria produced ESBL.

## Discussion

This is the largest study conducted to date to determine the prevalence of UTIs in pregnant women, and the resistance patterns of urine bacterial isolates were evaluated in pregnant and nonpregnant women in the capital city of Saudi Arabia. The study included 2,418 urine cultures, corresponding to 985 performed for pregnant women and 1,433 performed for nonpregnant women.

In this study, the overall prevalence of UTIs in pregnant women was 5% (95% CI 3.6–6.4). The predominant bacterial pathogen identified in women with UTIs was *E. coli* (134, 54.9%), followed by *K. pneumoniae (54.9%)* (48, 19.6%). Our findings showed that the prevalence of UTIs in pregnant women was lower than that reported locally and globally; however, it is comparable to the range (1.9–9.5%) reported by the Infectious Diseases Society of America (IDSA) [19]. A plausible reason for the low UTI prevalence in this study is that our patient population was relatively healthy, was educated according to university hospital eligibility criteria and had access to standard prenatal care as managed by the institution.

In the present study, the most common causative agent in both pregnant and nonpregnant women was *E. coli* (54.9%), followed by *K. pneumoniae* (19.6%). Interestingly, according to published local studies, the most common pathogens causing UTIs in pregnant women were *E. coli*, Staphylococcus, and Candida species, which are indications of fecal contamination and poor personal hygiene. This result provided support for the hypothesis that our patient population was relatively healthy and educated.

According to our findings, nitrofurantoin and amoxicillin-clavulanic acid should be recommended for use as an empirical therapy for UTIs in pregnant and nonpregnant women because bacteria have the least amount of resistance to these drugs, considering that nitrofurantoin should be avoided in the 3rd trimester of pregnancy [21]. On the other hand, bacteria had the greatest resistance to ampicillin, trimethoprim-sulfamethoxazole, and ciprofloxacin. To improve appropriate antibiotic use, antimicrobial stewardship teams should evaluate susceptibility patterns regularly as new resistant strains emerge and empirical therapy changes accordingly to prevent the misuse or overuse of antibiotics.

The limitations of this study include the retrospective single-center nature of this analysis. The results were dependent on accurate and complete documentation in the electronic medical records. The size and health status of our cohort may have been inadequately powered to detect significant findings and other associated risk factors for UTIs. Moreover, the policies and procedures practiced at our institution may not be reflective of other institutional practices. Future research involving molecular testing and comparisons of genes related to antibiotic resistance or biofilm formation is recommended.

## Conclusion

Pregnant women had a lower rate of UTIs in this study than did pregnant women in previous studies of a similar nature, which might be explained by the inclusion of healthy, educated women who had access to standard prenatal education and health care facilities at a university hospital.

## Data Availability

The datasets used and analyzed during the current study are available from the corresponding author on reasonable request, and contact information for the corresponding author have been provided.

## Abbreviations

UTIs: Urinary tract infections
KAAUH: King Abdullah bin Abdul-Aziz University Hospital
µL: Microliter
CLSI: Clinical and Laboratory Standards Institute
ESBL: Extended spectrum beta-lactamase
ASB: Asymptomatic bacteriuria
CFU: Colony forming units
mL: Milliliters
SPSS: Statistical Package for Social Sciences
IQRs: Interquartile ranges
*E. coli*: Escherichia coli
*K. pneumonia*: *Klebsiella pneumoniae*
IDSA: Infectious Diseases Society of America
